# Serum Paraoxonase/Arylesterase Activity and Oxidative Stress Status in Children with Metabolic Syndrome

**DOI:** 10.4274/jcrpe.1454

**Published:** 2014-09-05

**Authors:** Erdal Eren, Mahmut Abuhandan, Abdullah Solmaz, Abdullah Taşkın

**Affiliations:** 1 Harran University, Faculty of Medicine, Department of Pediatric Endocrinology, Şanlıurfa, Turkey; 2 Harran University, Faculty of Medicine, Department of Pediatrics, Şanlıurfa, Turkey; 3 Harran University, Faculty of Medicine, Department of Biochemistry, Şanlıurfa, Turkey

**Keywords:** metabolic syndrome, obesity, oxidative stress, paraoxonase

## Abstract

**Ob­jec­ti­ve:** This study aimed to measure paraoxonase/arylesterase activities and to evaluate the total oxidant and antioxidant capacities in obese children and in children with metabolic syndrome (MetS).

**Methods:** A total of 151 children of comparable ages (13.23±1.96 years, 13.45±1.85 years and 13.95±1.31 years) were enrolled in the study. Forty of these were children with MetS, 55 were obese and 56 were healthy controls. Diagnosis of the MetS was made according to International Diabetes Federation criteria. Paraoxonase/arylesterase activities were evaluated by using paraoxon and phenylacetate substrates. Total oxidant status (TOS) and total antioxidant status (TAS) were measured and oxidative stress index (OSI) was estimated by calculation.

**Results:** High levels of paraoxonase were detected in the obese group, whereas high levels of arylesterase were detected in both MetS and obese groups. Higher values for TOS, TAS and OSI were found in the MetS group (p<0.05).

**Conclusion:** Higher values of mean TOS and OSI in the MetS group than those in the control groups indicate that these parameters may be indicators of future risks such as atherosclerosis in patients with MetS.

## INTRODUCTION

Obesity is a global disease, which can be fatal and which usually has its start in childhood. Metabolic syndrome (MetS), which is encountered in adults, has also been observed in children in recent years. It has been reported that cardiovascular disease risk is higher in children with MetS (1). The abdominal distribution of adipose tissue is an important indicator as intraabdominal adiposity is related to insulin resistance and dyslipidemia and this type of fat distribution is also significant for MetS (2). Hyperglycemia also contributes to the development of lesions in the coronary arteries and aorta (3). In addition, hyperinsulinemia and deteriorated glycemic control are reported to cause an increase in oxidative stress (4). Oxidative stress is also a factor affecting the pathophysiology of MetS (5,6). Paraoxonase (PON)-1 (having PON and arylesterase activities) is an antioxidant enzyme related to high-density lipoproteins (HDL). PON is a glycoprotein synthesized from the liver. Experimental studies indicate that PON prevents atherosclerosis (7). PON1 activity is defined to be low in adults with MetS (8). Studies on adults have established that there is a relationship between coronary heart disease and PON1 activity (9,10). There are a few studies in children on the relationship between PON and MetS. Our aim was to evaluate PON1 activity as well as the total oxidant and antioxidant levels in children.

## METHODS

The study was performed on patients attending the pediatric endocrinology outpatient clinic of our hospital. Patients with body mass index (BMI) values over the 95th percentile for age and gender were accepted as obese (11). Obese cases were divided into two groups as those with MetS and those without MetS. Patients with MetS were defined as subjects showing three or more of the following parameters: abdominal obesity (increased waist circumference, increased waist-height ratio), hypertension, hyperglycemia-insulin resistance, high triglyceride (TG) levels, low HDL levels (12). The control group was composed of healthy age- and gender-matched participants. Subjects with systemic diseases and syndromic diseases were excluded from the study. A total of 151 cases (83 females, 68 males) were included in the study. Data from 40 cases (22 females, 18 males) with MetS, 55 cases (27 females, 28 males) with obesity and 56 cases (34 females, 22 males) in the control group were evaluated. Height, weight, BMI and waist circumference/height ratio values of the participants were recorded. Standard deviation scores (SDS) for height, weight and BMI were calculated (13). Informed consent was obtained from the parents of all the children. The study was approved by the Local Ethics Committee of Harran University Medical School.

Blood samples were taken from all cases in the morning between 8:00 and 9:00 a.m. following an overnight fast. The blood samples were centrifuged at 3500 rpm for 10 minutes, then the formed elements were discarded and the serum samples stored at -80°C. Measurements included glucose, insulin, alanine transaminase (ALT), plasma TG, total cholesterol, low-density lipoprotein (LDL), HDL, high-sensitivity C-reactive protein (hsCRP) levels, paraoxonase/arylesterase activity, as well as total oxidant and antioxidant capacities. homeostasis model assessment insulin ratioHOMA-IR index (glucose x insulin/405) was used for the detection of insulin resistance. The total antioxidant status (TAS) of plasma was determined using a novel automated measurement method (Abbott Aeroset, Abbott Diagnostics, Abbott Park, IL, USA), developed by Erel (14). In this method, the most potent biological radical, hydroxyl radical, is produced. In the assay, the ferrous ion solution, which is present in reagent 1 [o-dianisidine (10 mM), ferrous ion (45 AM) in the Clark and Lubs solution (75 mM, pH 1.8] is mixed with hydrogen peroxide, which is present in reagent 2 [H2O2 (7.5 mM) in the Clark and Lubs solution]. The sequentially produced radicals such as brown-colored dianisidinyl radical cation, formed by the hydroxyl radical, are also potent radicals. Using this method, the antioxidative effect of the sample against the potent free radical reactions initiated by the produced hydroxyl radical is measured. The assay has excellent precision values of lower than 3%. The results were expressed as mmol Trolox Equivalent L-1.

Total oxidant status (TOS) of plasma was determined using a novel automated measurement method, developed by Erel (15). Oxidants present in the sample oxidized the ferrous ion-o-dianisidine complex to ferric ion. The oxidation reaction was enhanced by glycerol molecules which were abundantly present in the reaction medium. The ferric ion produced a colored complex with xylenol orange in an acidic medium. The color intensity, which can be measured spectrophotometrically, was related to the total amount of oxidant molecules present in the sample. The assay was calibrated with hydrogen peroxide and the results were expressed in terms of micromolar hydrogen peroxide equivalent per liter (mmol H2O2 Equivalent L-1). 

The ratio percentage of TOS level to TAS level gave the oxidative stress index (OSI), an indicator of the degree of oxidative stress (15). 

Plasma TG, total cholesterol, LDL and HDL levels were measured by an automated chemistry analyzer (Aeroset, Abbott, USA) using Abbott commercial kits. Serum hsCRP level was measured using an available commercial kit (Roche). 

Paraoxonase/arylesterase activities were measured using paraoxon and phenylacetate substrates. The rate of paraoxonhydrolysis (diethyl-p-nitrophenylphosphate) was measured by monitoring the increase of absorbance at 412 nm at 37 °C. The amount of generated pnitrophenol was calculated from the molar absorptivity coefficient at pH 8, which was 17.000 M-1 cm-1. Paraoxonase activity was expressed as U/L serum. Phenylacetate was used as a substrate to measure the arylesterase activity. Enzymatic activity was calculated from the molar absorptivity coefficient of the produced phenol, 1310 M-1 cm-1. One unit of arylesterase activity was defined as 1 μmol phenol generated/min under the above conditions and expressed as U/L serum (16). Paraoxonase phenotype distribution was determined by a double substrate method that measures the ratio of paraoxonase activity (with 1 M NaCl in the assay) to arylesterase activity, using phenylacetate (17).

**Statistical Analysis**

The data were analyzed using SPSS (Statistical Package for the Social Sciences, version 11.5 for Windows, SPSS® Inc, Chicago, IL). The distribution of parametric variables was assessed with one-sample Kolmogorov-Smirnov test when all parametric variables were not distributed normally. The results were presented as mean±SD. The Kruskal-Wallis and chi-square tests were used for univariate analysis. A two-tailed p-value of less than 0.05 was considered statistically significant.

## DISCUSSION

Oxidative stress is believed to be the trigger of many diseases. Obesity and/or MetS adversely affect the oxidant system. Low HDL level, one of the criteria for MetS, is a significant marker of a weak antioxidant defense system. Oxidant effect, which may be caused by LDL, is counteracted by the antioxidant effect of HDL. Moreover, HDL has anti-inflammatory characteristics, so it suppresses cytokine originated endothelial cell adhesion molecules (17). The antioxidant quality of HDL is related to serum PON1. PON1 activity has been defined to be decreased in adult obese patients (18). Garin et al (8) state that PON1 activity is lower in subjects with MetS than in controls. Tabur et al (19) have reported that there is no difference in PON1 activity between subjects with MetS and obese subjects with no MetS. Studies indicate that PON1 activity decreases with age (20). Studies related to PON and obesity have been performed mostly in adults. However, studies also indicate that PON activity is low in obese children (21,22). Ferre et al (23) have reported that PON1 may play a role in the onset and development of metabolic alterations in childhood obesity. We found elevated PON1 activity in both MetS and obese groups, which was statistically significant between the obese and control groups. Moreover, the arylesterase level, an indicator of PON1 activity, was detected as markedly high both in MetS and obese groups compared with the control group. This finding suggests that in children, PON1 prevents lipid peroxidation by playing a preventive role against the oxidative modification of LDL cholesterol. It thus slows the atherosclerosis progression with its antioxidant and anti-inflammatory effects. It was also observed that PON1 showed antioxidant characteristics against lipid peroxidation caused by free radicals on cellular membranes and lipoproteins (24). 

Lee et al (25) state that antioxidant capacity is related to HDL2 in patients with decreased HDL cholesterol levels when compared with the control groups. These authors report that HDL2-related PON activity increases by nearly three-fold in such patients and that the antioxidant capacity of PON could be related to HDL2 and HDL3. Despite having hypertriglyceridemia and hypercholesterolemia, patients may also have potent PON1 activity and this finding shows that the HDL cholesterol level is not correlated with PON activity. The results of our study were consistent with these findings.

In a study on obese children with and without MetS to evaluate oxidant systems, it has been established that reactive oxidant metabolites are markedly higher in both groups when compared with the control group (26). Our study showed that the fasting insulin level and insulin resistance were significantly high in the MetS and obese groups and that the TOS value in the MetS group was higher than that in both the control and obese groups. Similarly, OSI values were markedly higher in the MetS group. These results indicate that hyperinsulinemia and deteriorated glycemic control contribute to the increase of oxidative stress levels in MetS. Similar to many other diseases, oxidative stress may play a role in the pathogenesis of MetS.

Studies have shown that central obesity is related to the decreased antioxidant capacity in adults. In adult obese cases with or without MetS, TAS levels are lower, while PON and arylesterase levels remain unchanged (19). Another study reveals that children with MetS have high levels of oxidative stress but that the antioxidant stress remains unchanged (27). Pirgon et al (28) state that oxidative stress levels are higher and TAS levels are lower in obese children with non-alcoholic hepatic steatosis, compared with the control group. In contrast, Torun el at (29) found increased TAS levels in obese children. We also found higher TAS levels in the MetS group, a finding which can be evaluated as a potent response of the organism against the oxidant system. Oxidative stress may be an early predictor of atherosclerosis progression. As age increases, the decrease in TAS levels can be effective on atherosclerosis progression. The increased TAS and TOS levels in the MetS group of our study indicate that oxidant stress levels are high in the obese subjects and that the antioxidant system worked to provide the equilibrium. Again, marked increases of PON in the obese group, as well as increased arylesterase levels in both obese and MetS groups are findings which indicate the role of PON1 activity in the antioxidant system.

CRP is the acute phase reactant, secreted from the liver in reaction to inflammation. It is evident that there are marked increases in CRP and oxidative stress levels in obese children with MetS, and these could pose a risk for heart diseases (30,31). A Norwegian study on 2 300 cases with participants between the ages of 9 and 15 years reports a positive correlation between waist circumference and hsCRP (32). While Kitsios et al (33) report that hsCRP levels are higher in obese cases and that the levels are similar in the MetS cases, in another study in Spain, higher hsCRP levels were reported in MetS cases as compared to obese cases (34). In our study, hsCRP levels were found to be significantly higher in the MetS and obese groups compared with the control group. However, there was no difference in hsCRP levels between the MetS and obese groups.

In conclusion, our study showed that oxidant and antioxidant capacities, PON1 activity and hsCRP were high in children with MetS and also in obese children. When compared with studies on adults, the high antioxidant and PON1 levels indicated that protective mechanisms worked. Apparently, the activity of the antioxidant system decreases with age, while the atherosclerotic process is accelerated. Our study also showed that the markers of obesity in humans are initiated during the childhood period. 

## Figures and Tables

**Table 1:**
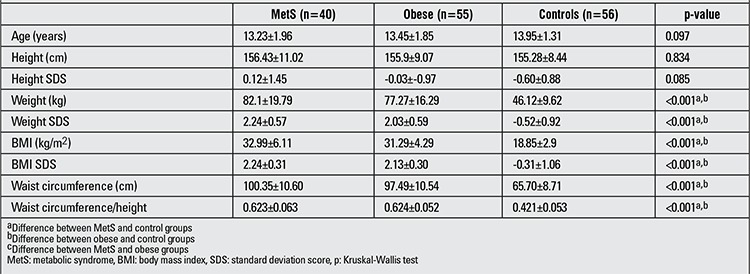
Auxological data of the cases in the three groups

**Table 2:**
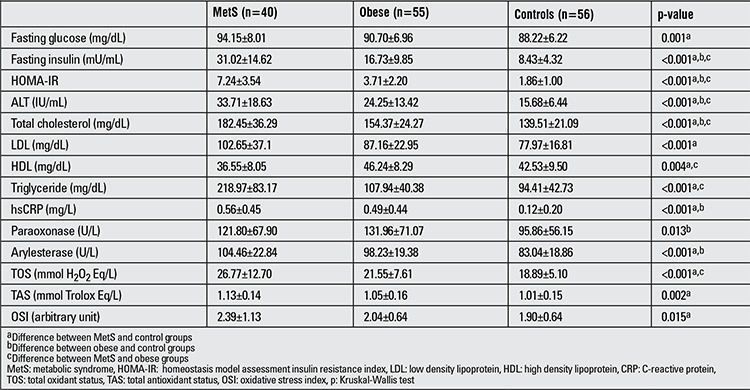
Laboratory data of the cases in the three groups

**Figure 1:**
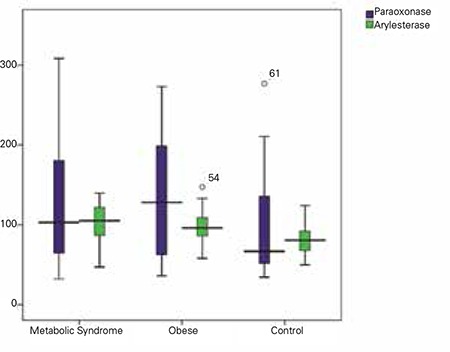
Changes in paraoxonase/arylesterase activity in the three groups
